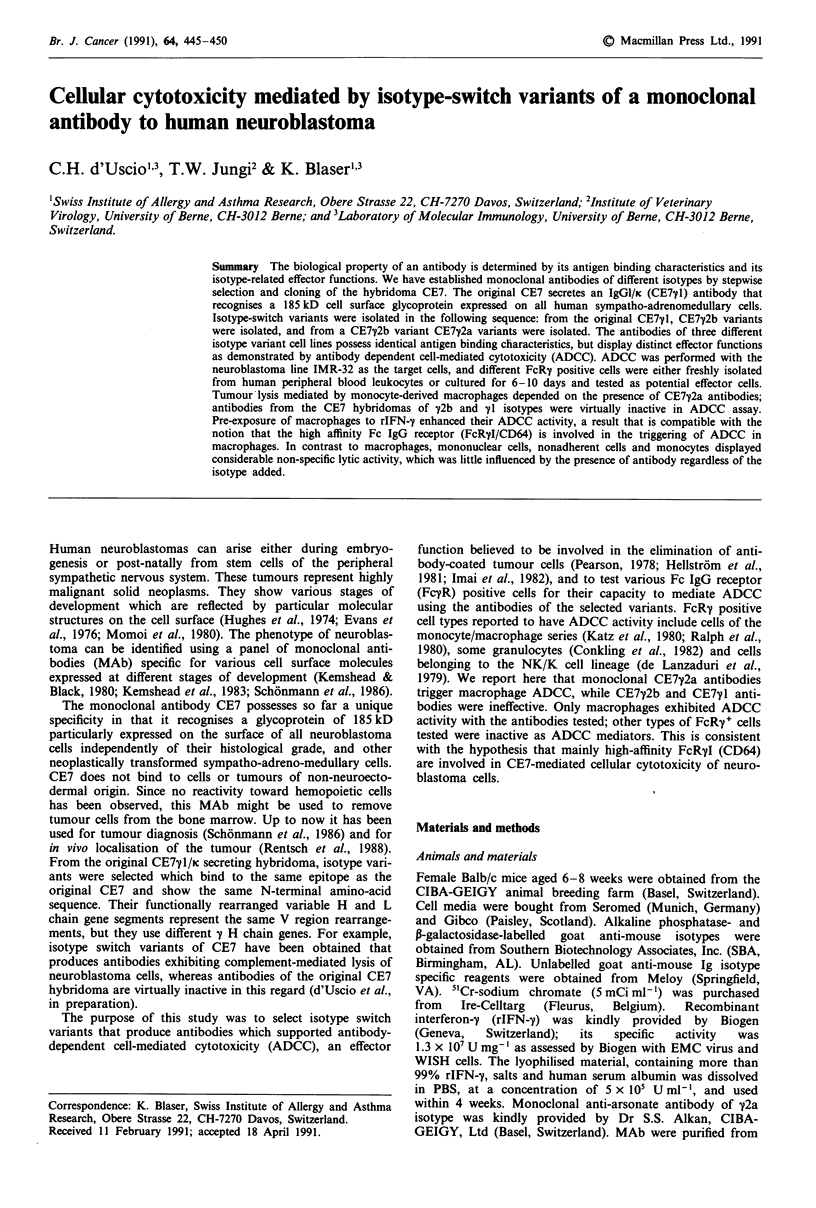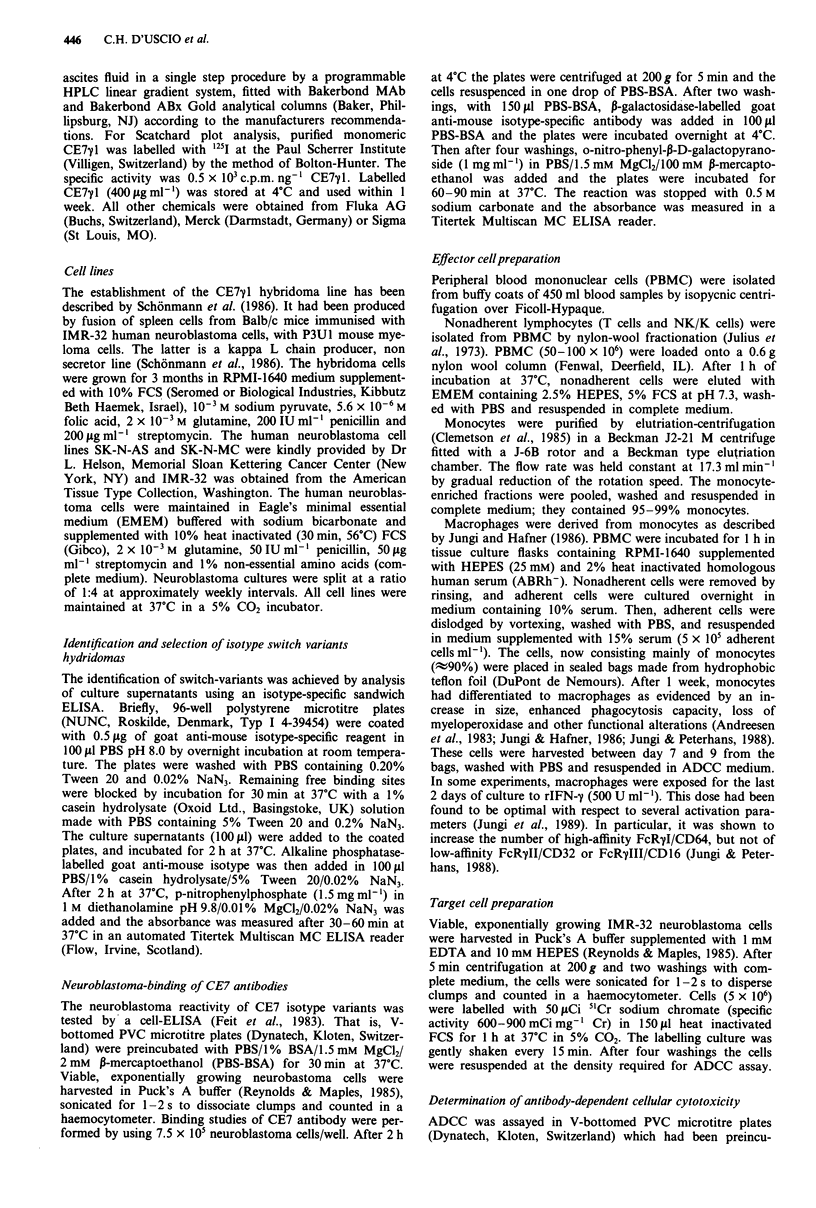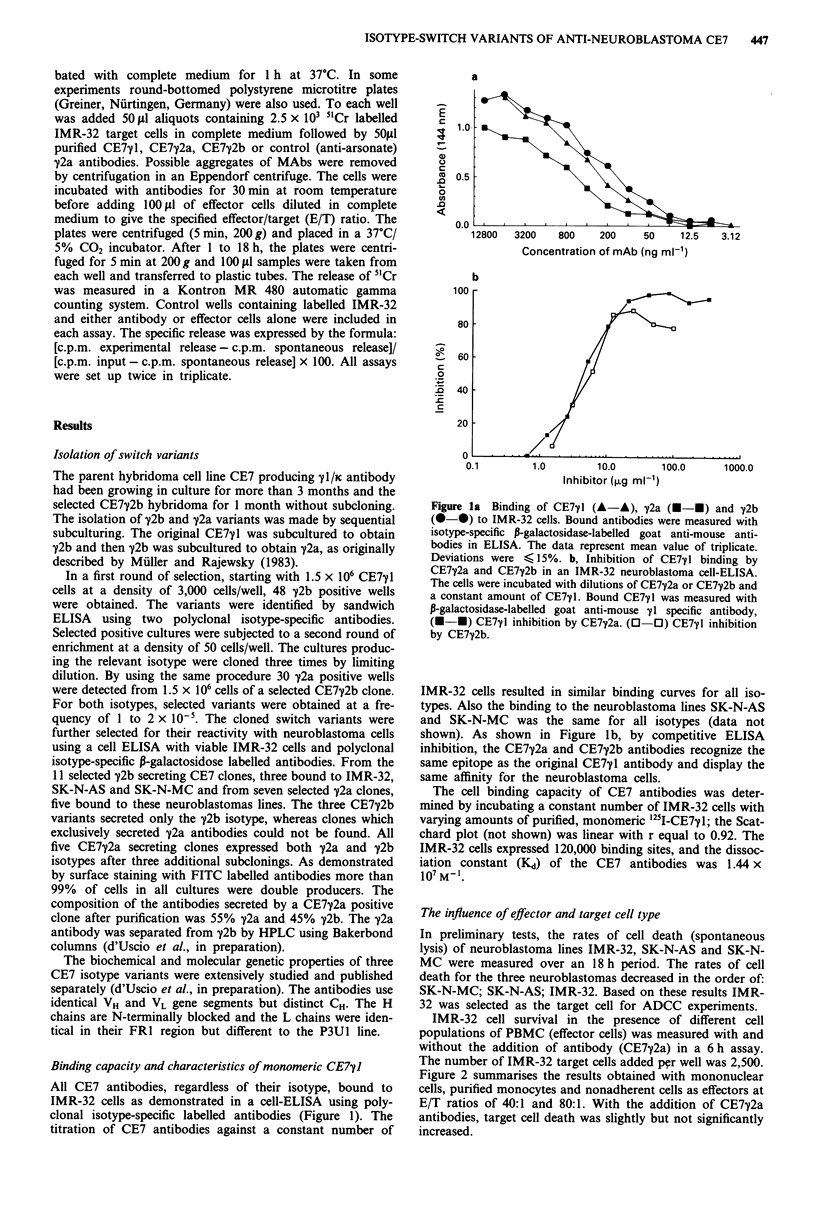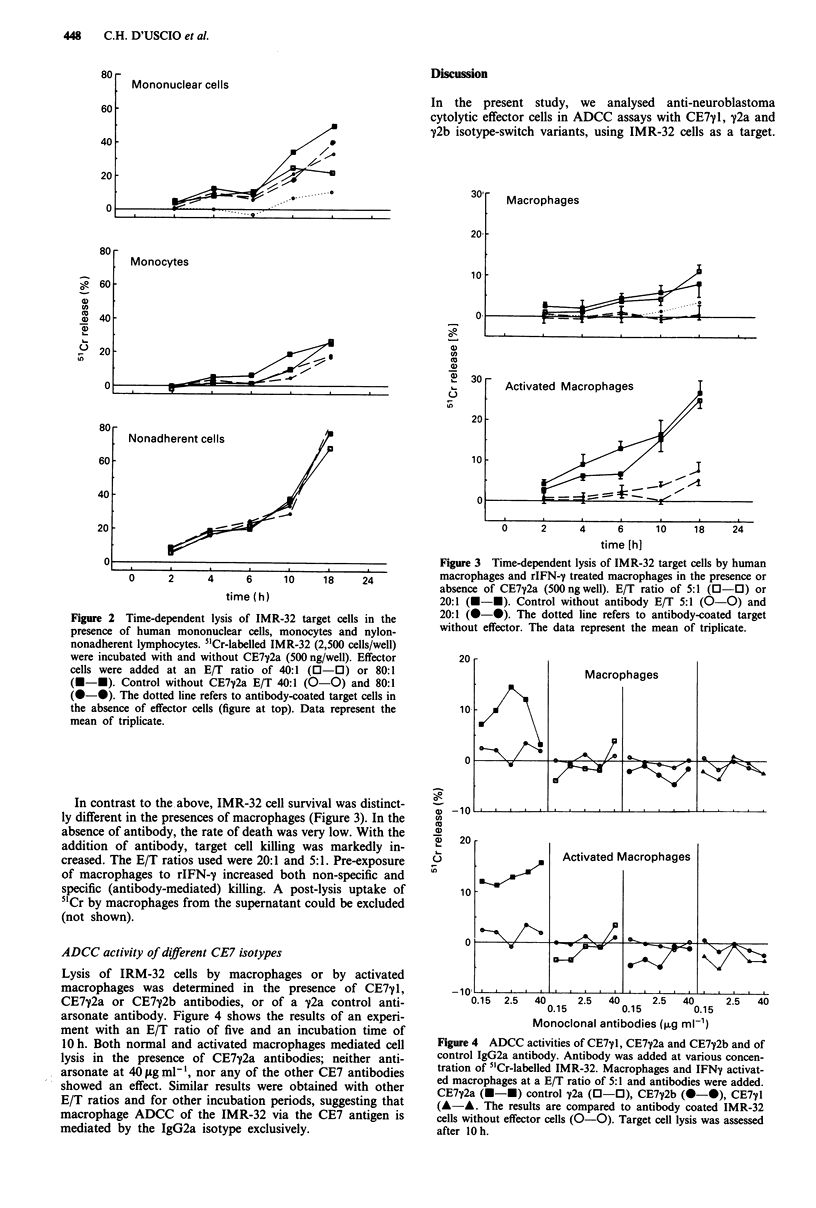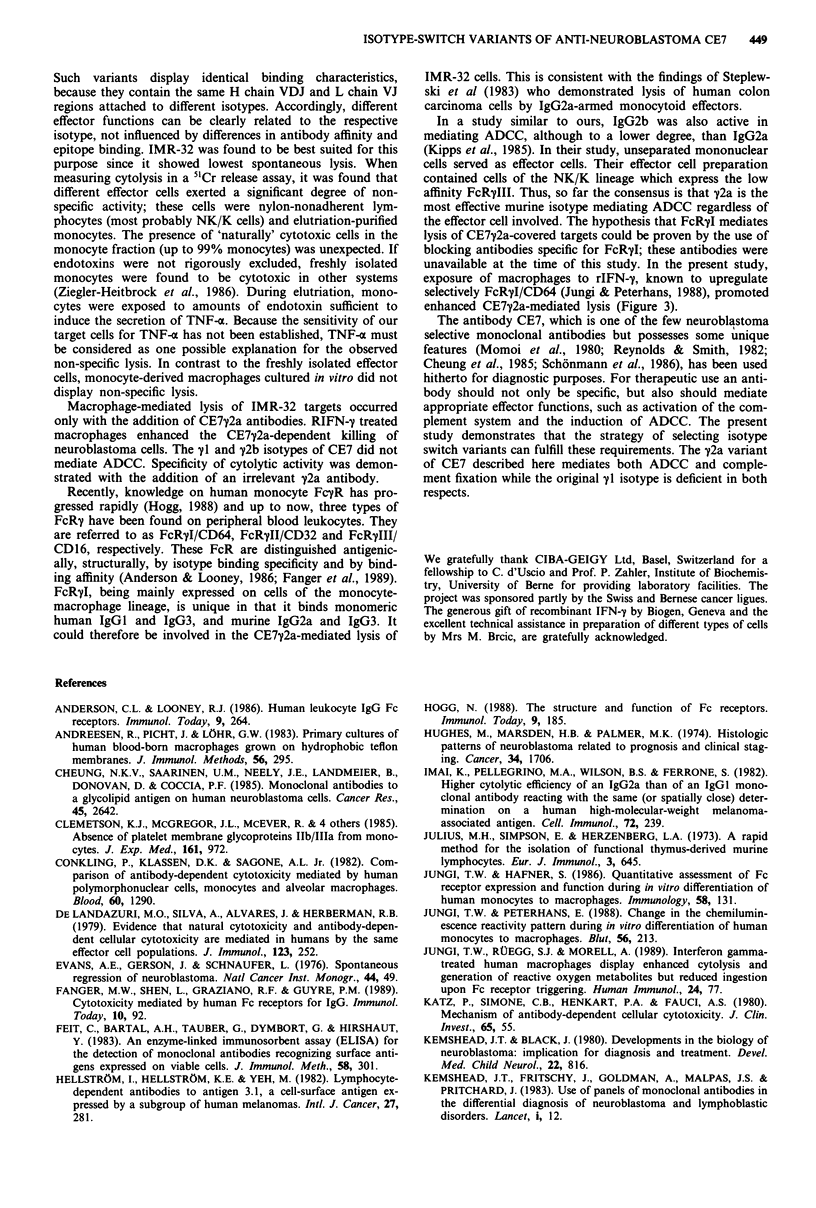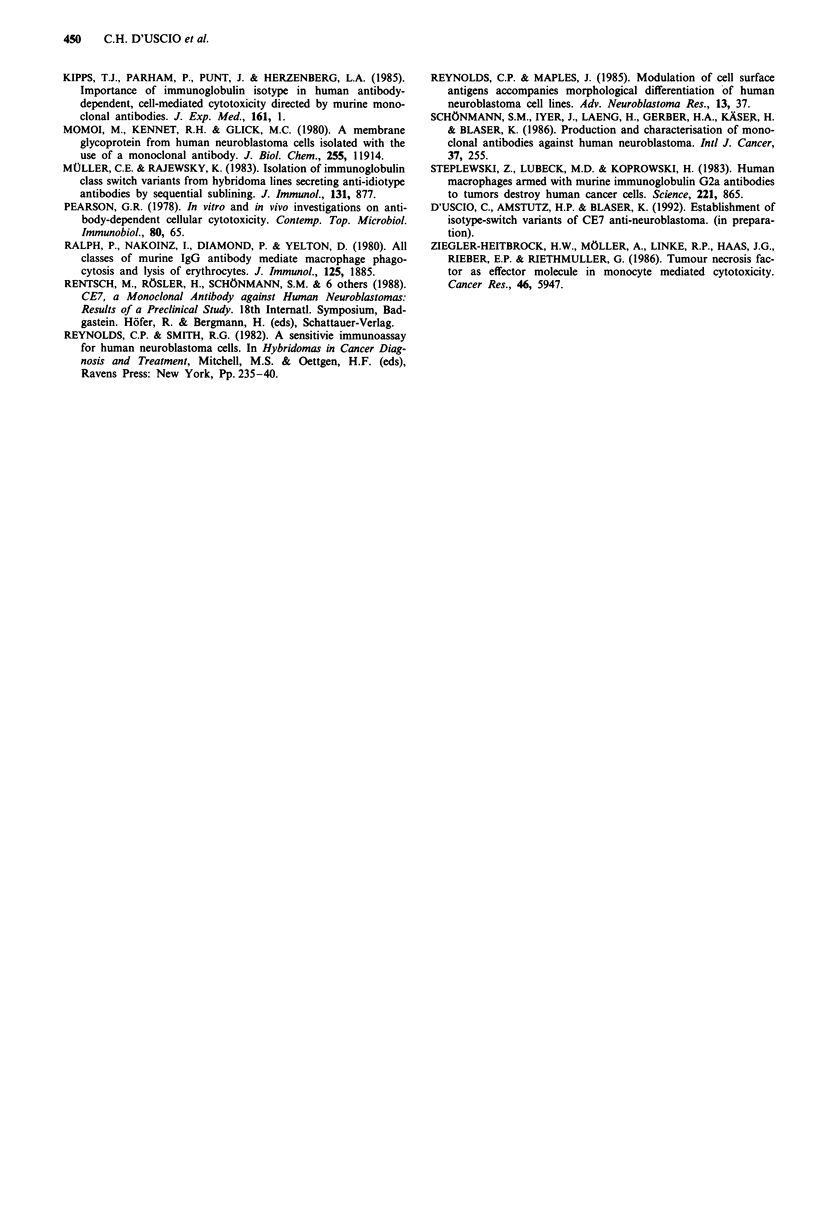# Cellular cytotoxicity mediated by isotype-switch variants of a monoclonal antibody to human neuroblastoma.

**DOI:** 10.1038/bjc.1991.329

**Published:** 1991-09

**Authors:** C. H. d'Uscio, T. W. Jungi, K. Blaser

**Affiliations:** Swiss Institute of Allergy and Asthma Research, Davos.

## Abstract

The biological property of an antibody is determined by its antigen binding characteristics and its isotype-related effector functions. We have established monoclonal antibodies of different isotypes by stepwise selection and cloning of the hybridoma CE7. The original CE7 secretes an IgG1/kappa (CE7 gamma 1) antibody that recognises a 185 kD cell surface glycoprotein expressed on all human sympatho-adrenomedullary cells. Isotype-switch variants were isolated in the following sequence: from the original CE7 gamma 1, CE7 gamma 2b variants were isolated, and from a CE7 gamma 2b variant CE7 gamma 2a variants were isolated. The antibodies of three different isotype variant cell lines possess identical antigen binding characteristics, but display distinct effector functions as demonstrated by antibody dependent cell-mediated cytotoxicity (ADCC). ADCC was performed with the neuroblastoma line IMR-32 as the target cells, and different FcR gamma positive cells were either freshly isolated from human peripheral blood leukocytes or cultured for 6-10 days and tested as potential effector cells. Tumour lysis mediated by monocyte-derived macrophages depended on the presence of CE7 gamma 2a antibodies; antibodies from the CE7 hybridomas of gamma 2b and gamma 1 isotypes were virtually inactive in ADCC assay. Pre-exposure of macrophages to rIFN-gamma enhanced their ADCC activity, a result that is compatible with the notion that the high affinity Fc IgG receptor (FcR gamma I/CD64) is involved in the triggering of ADCC in macrophages. In contrast to macrophages, mononuclear cells, nonadherent cells and monocytes displayed considerable non-specific lytic activity, which was little influenced by the presence of antibody regardless of the isotype added.


Br. J. Cancer (1991), 64, 445–450 © Macmillan Press Ltd., 1991

### Cellular cytotoxicity mediated by isotype-switch variants of a monoclonal antibody to human neuroblastoma

C.H. d'Uscio[1,3], T.W. Jungi[2] & K. Blaser[1,3]

[1]*Swiss Institute of Allergy and Asthma Research, Obere Strasse 22, CH-7270 Davos, Switzerland;* [2]*Institute of Veterinary Virology, University of Berne, CH-3012 Berne; and* [3]*Laboratory of Molecular Immunology, University of Berne, CH-3012 Berne, Switzerland.*

**Summary** The biological property of an antibody is determined by its antigen binding characteristics and its isotype-related effector functions. We have established monoclonal antibodies of different isotypes by stepwise selection and cloning of the hybridoma CE7. The original CE7 secretes an IgGl/κ (CE7γ1) antibody that recognises a 185 kD cell surface glycoprotein expressed on all human sympatho-adrenomedullary cells. Isotype-switch variants were isolated in the following sequence: from the original CE7γ1, CE7γ2b variants were isolated, and from a CE7γ2b variant CE7γ2a variants were isolated. The antibodies of three different isotype variant cell lines possess identical antigen binding characteristics, but display distinct effector functions as demonstrated by antibody dependent cell-mediated cytotoxicity (ADCC). ADCC was performed with the neuroblastoma line IMR-32 as the target cells, and different FcRγ positive cells were either freshly isolated from human peripheral blood leukocytes or cultured for 6–10 days and tested as potential effector cells. Tumour lysis mediated by monocyte-derived macrophages depended on the presence of CE7γ2a antibodies; antibodies from the CE7 hybridomas of γ2b and γ1 isotypes were virtually inactive in ADCC assay. Pre-exposure of macrophages to rIFN-γ enhanced their ADCC activity, a result that is compatible with the notion that the high affinity Fc IgG receptor (FcRγI/CD64) is involved in the triggering of ADCC in macrophages. In contrast to macrophages, mononuclear cells, nonadherent cells and monocytes displayed considerable non-specific lytic activity, which was little influenced by the presence of antibody regardless of the isotype added.

Human neuroblastomas can arise either during embryogenesis or post-natally from stem cells of the peripheral sympathetic nervous system. These tumours represent highly malignant solid neoplasms. They show various stages of development which are reflected by particular molecular structures on the cell surface (Hughes *et al.*, 1974; Evans *et al.*, 1976; Momoi *et al.*, 1980). The phenotype of neuroblastoma can be identified using a panel of monoclonal antibodies (MAb) specific for various cell surface molecules expressed at different stages of development (Kemshead & Black, 1980; Kemshead *et al.*, 1983; Schönmann *et al.*, 1986).

The monoclonal antibody CE7 possesses so far a unique specificity in that it recognises a glycoprotein of 185 kD particularly expressed on the surface of all neuroblastoma cells independently of their histological grade, and other neoplastically transformed sympatho-adreno-medullary cells. CE7 does not bind to cells or tumours of non-neuroectodermal origin. Since no reactivity toward hemopoietic cells has been observed, this MAb might be used to remove tumour cells from the bone marrow. Up to now it has been used for tumour diagnosis (Schönmann *et al.*, 1986) and for *in vivo* localisation of the tumour (Rentsch *et al.*, 1988). From the original CE7γ1/κ secreting hybridoma, isotype variants were selected which bind to the same epitope as the original CE7 and show the same N-terminal amino-acid sequence. Their functionally rearranged variable H and L chain gene segments represent the same V region rearrangements, but they use different γ H chain genes. For example, isotype switch variants of CE7 have been obtained that produces antibodies exhibiting complement-mediated lysis of neuroblastoma cells, whereas antibodies of the original CE7 hybridoma are virtually inactive in this regard (d'Uscio *et al.*, in preparation).

The purpose of this study was to select isotype switch variants that produce antibodies which supported antibody-dependent cell-mediated cytotoxicity (ADCC), an effector function believed to be involved in the elimination of antibody-coated tumour cells (Pearson, 1978; Hellström *et al.*, 1981; Imai *et al.*, 1982), and to test various Fc IgG receptor (FcγR) positive cells for their capacity to mediate ADCC using the antibodies of the selected variants. FcRγ positive cell types reported to have ADCC activity include cells of the monocyte/macrophage series (Katz *et al.*, 1980; Ralph *et al.*, 1980), some granulocytes (Conkling *et al.*, 1982) and cells belonging to the NK/K cell lineage (de Lanzaduri *et al.*, 1979). We report here that monoclonal CE7γ2a antibodies trigger macrophage ADCC, while CE7γ2b and CE7γ1 antibodies were ineffective. Only macrophages exhibited ADCC activity with the antibodies tested; other types of $FcR\gamma^+$ cells tested were inactive as ADCC mediators. This is consistent with the hypothesis that mainly high-affinity FcRγI (CD64) are involved in CE7-mediated cellular cytotoxicity of neuroblastoma cells.

### Materials and methods

### *Animals and materials*

Female Balb/c mice aged 6–8 weeks were obtained from the CIBA-GEIGY animal breeding farm (Basel, Switzerland). Cell media were bought from Seromed (Munich, Germany) and Gibco (Paisley, Scotland). Alkaline phosphatase- and β-galactosidase-labelled goat anti-mouse isotypes were obtained from Southern Biotechnology Associates, Inc. (SBA, Birmingham, AL). Unlabelled goat anti-mouse Ig isotype specific reagents were obtained from Meloy (Springfield, VA). $^{51}$Cr-sodium chromate ($5\,mCi\,ml^{-1}$) was purchased from Ire-Celltarg (Fleurus, Belgium). Recombinant interferon-γ (rIFN-γ) was kindly provided by Biogen (Geneva, Switzerland); its specific activity was $1.3 \times 10^7\,U\,mg^{-1}$ as assessed by Biogen with EMC virus and WISH cells. The lyophilised material, containing more than 99% rIFN-γ, salts and human serum albumin was dissolved in PBS, at a concentration of $5 \times 10^5\,U\,ml^{-1}$, and used within 4 weeks. Monoclonal anti-arsonate antibody of γ2a isotype was kindly provided by Dr S.S. Alkan, CIBA-GEIGY, Ltd (Basel, Switzerland). MAb were purified from

Correspondence: K. Blaser, Swiss Institute of Allergy and Asthma Research, Obere Strasse 22, CH-7270 Davos, Switzerland.
Received 11 February 1991; accepted 18 April 1991.

ascites fluid in a single step procedure by a programmable HPLC linear gradient system, fitted with Bakerbond MAb and Bakerbond ABx Gold analytical columns (Baker, Phillipsburg, NJ) according to the manufacturers recommendations. For Scatchard plot analysis, purified monomeric CE7γ1 was labelled with $^{125}I$ at the Paul Scherrer Institute (Villigen, Switzerland) by the method of Bolton-Hunter. The specific activity was $0.5 \times 10^3$ c.p.m. $ng^{-1}$ CE7γ1. Labelled CE7γ1 (400 μg $ml^{-1}$) was stored at 4°C and used within 1 week. All other chemicals were obtained from Fluka AG (Buchs, Switzerland), Merck (Darmstadt, Germany) or Sigma (St Louis, MO).

*Cell lines*

The establishment of the CE7γ1 hybridoma line has been described by Schönmann *et al.* (1986). It had been produced by fusion of spleen cells from Balb/c mice immunised with IMR-32 human neuroblastoma cells, with P3U1 mouse myeloma cells. The latter is a kappa L chain producer, non secretor line (Schönmann *et al.*, 1986). The hybridoma cells were grown for 3 months in RPMI-1640 medium supplemented with 10% FCS (Seromed or Biological Industries, Kibbutz Beth Haemek, Israel), $10^{-3}$ M sodium pyruvate, $5.6 \times 10^{-6}$ M folic acid, $2 \times 10^{-3}$ M glutamine, 200 IU $ml^{-1}$ penicillin and 200 μg $ml^{-1}$ streptomycin. The human neuroblastoma cell lines SK-N-AS and SK-N-MC were kindly provided by Dr L. Helson, Memorial Sloan Kettering Cancer Center (New York, NY) and IMR-32 was obtained from the American Tissue Type Collection, Washington. The human neuroblastoma cells were maintained in Eagle's minimal essential medium (EMEM) buffered with sodium bicarbonate and supplemented with 10% heat inactivated (30 min, 56°C) FCS (Gibco), $2 \times 10^{-3}$ M glutamine, 50 IU $ml^{-1}$ penicillin, 50 μg $ml^{-1}$ streptomycin and 1% non-essential amino acids (complete medium). Neuroblastoma cultures were split at a ratio of 1:4 at approximately weekly intervals. All cell lines were maintained at 37°C in a 5% $CO_2$ incubator.

*Identification and selection of isotype switch variants hydridomas*

The identification of switch-variants was achieved by analysis of culture supernatants using an isotype-specific sandwich ELISA. Briefly, 96-well polystyrene microtitre plates (NUNC, Roskilde, Denmark, Typ I 4-39454) were coated with 0.5 μg of goat anti-mouse isotype-specific reagent in 100 μl PBS pH 8.0 by overnight incubation at room temperature. The plates were washed with PBS containing 0.20% Tween 20 and 0.02% $NaN_3$. Remaining free binding sites were blocked by incubation for 30 min at 37°C with a 1% casein hydrolysate (Oxoid Ltd., Basingstoke, UK) solution made with PBS containing 5% Tween 20 and 0.2% $NaN_3$. The culture supernatants (100 μl) were added to the coated plates, and incubated for 2 h at 37°C. Alkaline phosphatase-labelled goat anti-mouse isotype was then added in 100 μl PBS/1% casein hydrolysate/5% Tween 20/0.02% $NaN_3$. After 2 h at 37°C, p-nitrophenylphosphate (1.5 mg $ml^{-1}$) in 1 M diethanolamine pH 9.8/0.01% $MgCl_2$/0.02% $NaN_3$ was added and the absorbance was measured after 30–60 min at 37°C in an automated Titertek Multiscan MC ELISA reader (Flow, Irvine, Scotland).

*Neuroblastoma-binding of CE7 antibodies*

The neuroblastoma reactivity of CE7 isotype variants was tested by a cell-ELISA (Feit *et al.*, 1983). That is, V-bottomed PVC microtitre plates (Dynatech, Kloten, Switzerland) were preincubated with PBS/1% BSA/1.5 mM $MgCl_2$/2 mM β-mercaptoethanol (PBS-BSA) for 30 min at 37°C. Viable, exponentially growing neurobastoma cells were harvested in Puck's A buffer (Reynolds & Maples, 1985), sonicated for 1–2 s to dissociate clumps and counted in a haemocytometer. Binding studies of CE7 antibody were performed by using $7.5 \times 10^5$ neuroblastoma cells/well. After 2 h at 4°C the plates were centrifuged at 200 *g* for 5 min and the cells resuspenced in one drop of PBS-BSA. After two washings, with 150 μl PBS-BSA, β-galactosidase-labelled goat anti-mouse isotype-specific antibody was added in 100 μl PBS-BSA and the plates were incubated overnight at 4°C. Then after four washings, o-nitro-phenyl-β-D-galactopyranoside (1 mg $ml^{-1}$) in PBS/1.5 mM $MgCl_2$/100 mM β-mercaptoethanol was added and the plates were incubated for 60–90 min at 37°C. The reaction was stopped with 0.5 M sodium carbonate and the absorbance was measured in a Titertek Multiscan MC ELISA reader.

*Effector cell preparation*

Peripheral blood mononuclear cells (PBMC) were isolated from buffy coats of 450 ml blood samples by isopycnic centrifugation over Ficoll-Hypaque.

Nonadherent lymphocytes (T cells and NK/K cells) were isolated from PBMC by nylon-wool fractionation (Julius *et al.*, 1973). PBMC ($50-100 \times 10^6$) were loaded onto a 0.6 g nylon wool column (Fenwal, Deerfield, IL). After 1 h of incubation at 37°C, nonadherent cells were eluted with EMEM containing 2.5% HEPES, 5% FCS at pH 7.3, washed with PBS and resuspended in complete medium.

Monocytes were purified by elutriation-centrifugation (Clemetson *et al.*, 1985) in a Beckman J2-21 M centrifuge fitted with a J-6B rotor and a Beckman type elutriation chamber. The flow rate was held constant at 17.3 ml $min^{-1}$ by gradual reduction of the rotation speed. The monocyte-enriched fractions were pooled, washed and resuspended in complete medium; they contained 95–99% monocytes.

Macrophages were derived from monocytes as described by Jungi and Hafner (1986). PBMC were incubated for 1 h in tissue culture flasks containing RPMI-1640 supplemented with HEPES (25 mM) and 2% heat inactivated homologous human serum ($ABRh^-$). Nonadherent cells were removed by rinsing, and adherent cells were cultured overnight in medium containing 10% serum. Then, adherent cells were dislodged by vortexing, washed with PBS, and resuspended in medium supplemented with 15% serum ($5 \times 10^5$ adherent cells $ml^{-1}$). The cells, now consisting mainly of monocytes (≈90%) were placed in sealed bags made from hydrophobic teflon foil (DuPont de Nemours). After 1 week, monocytes had differentiated to macrophages as evidenced by an increase in size, enhanced phagocytosis capacity, loss of myeloperoxidase and other functional alterations (Andreesen *et al.*, 1983; Jungi & Hafner, 1986; Jungi & Peterhans, 1988). These cells were harvested between day 7 and 9 from the bags, washed with PBS and resuspended in ADCC medium. In some experiments, macrophages were exposed for the last 2 days of culture to rIFN-γ (500 U $ml^{-1}$). This dose had been found to be optimal with respect to several activation parameters (Jungi *et al.*, 1989). In particular, it was shown to increase the number of high-affinity FcRγI/CD64, but not of low-affinity FcRγII/CD32 or FcRγIII/CD16 (Jungi & Peterhans, 1988).

*Target cell preparation*

Viable, exponentially growing IMR-32 neuroblastoma cells were harvested in Puck's A buffer supplemented with 1 mM EDTA and 10 mM HEPES (Reynolds & Maples, 1985). After 5 min centrifugation at 200 *g* and two washings with complete medium, the cells were sonicated for 1–2 s to disperse clumps and counted in a haemocytometer. Cells ($5 \times 10^6$) were labelled with 50 μCi $^{51}Cr$ sodium chromate (specific activity 600–900 mCi $mg^{-1}$ Cr) in 150 μl heat inactivated FCS for 1 h at 37°C in 5% $CO_2$. The labelling culture was gently shaken every 15 min. After four washings the cells were resuspended at the density required for ADCC assay.

*Determination of antibody-dependent cellular cytotoxicity*

ADCC was assayed in V-bottomed PVC microtitre plates (Dynatech, Kloten, Switzerland) which had been preincubated with complete medium for 1 h at 37°C. In some experiments round-bottomed polystyrene microtitre plates (Greiner, Nürtingen, Germany) were also used. To each well was added 50 μl aliquots containing $2.5 \times 10^3$ $^{51}Cr$ labelled IMR-32 target cells in complete medium followed by 50μl purified CE7γ1, CE7γ2a, CE7γ2b or control (anti-arsonate) γ2a antibodies. Possible aggregates of MAbs were removed by centrifugation in an Eppendorf centrifuge. The cells were incubated with antibodies for 30 min at room temperature before adding 100 μl of effector cells diluted in complete medium to give the specified effector/target (E/T) ratio. The plates were centrifuged (5 min, 200 g) and placed in a 37°C/5% $CO_2$ incubator. After 1 to 18 h, the plates were centrifuged for 5 min at 200 g and 100 μl samples were taken from each well and transferred to plastic tubes. The release of $^{51}Cr$ was measured in a Kontron MR 480 automatic gamma counting system. Control wells containing labelled IMR-32 and either antibody or effector cells alone were included in each assay. The specific release was expressed by the formula: [c.p.m. experimental release − c.p.m. spontaneous release]/[c.p.m. input − c.p.m. spontaneous release] × 100. All assays were set up twice in triplicate.

### Results

### *Isolation of switch variants*

The parent hybridoma cell line CE7 producing γ1/κ antibody had been growing in culture for more than 3 months and the selected CE7γ2b hybridoma for 1 month without subcloning. The isolation of γ2b and γ2a variants was made by sequential subculturing. The original CE7γ1 was subcultured to obtain γ2b and then γ2b was subcultured to obtain γ2a, as originally described by Müller and Rajewsky (1983).

In a first round of selection, starting with $1.5 \times 10^6$ CE7γ1 cells at a density of 3,000 cells/well, 48 γ2b positive wells were obtained. The variants were identified by sandwich ELISA using two polyclonal isotype-specific antibodies. Selected positive cultures were subjected to a second round of enrichment at a density of 50 cells/well. The cultures producing the relevant isotype were cloned three times by limiting dilution. By using the same procedure 30 γ2a positive wells were detected from $1.5 \times 10^6$ cells of a selected CE7γ2b clone. For both isotypes, selected variants were obtained at a frequency of 1 to $2 \times 10^{-5}$. The cloned switch variants were further selected for their reactivity with neuroblastoma cells using a cell ELISA with viable IMR-32 cells and polyclonal isotype-specific β-galactosidose labelled antibodies. From the 11 selected γ2b secreting CE7 clones, three bound to IMR-32, SK-N-AS and SK-N-MC and from seven selected γ2a clones, five bound to these neuroblastomas lines. The three CE7γ2b variants secreted only the γ2b isotype, whereas clones which exclusively secreted γ2a antibodies could not be found. All five CE7γ2a secreting clones expressed both γ2a and γ2b isotypes after three additional subclonings. As demonstrated by surface staining with FITC labelled antibodies more than 99% of cells in all cultures were double producers. The composition of the antibodies secreted by a CE7γ2a positive clone after purification was 55% γ2a and 45% γ2b. The γ2a antibody was separated from γ2b by HPLC using Bakerbond columns (d'Uscio *et al.*, in preparation).

The biochemical and molecular genetic properties of three CE7 isotype variants were extensively studied and published separately (d'Uscio *et al.*, in preparation). The antibodies use identical $V_H$ and $V_L$ gene segments but distinct $C_H$. The H chains are N-terminally blocked and the L chains were identical in their FR1 region but different to the P3U1 line.

### *Binding capacity and characteristics of monomeric CE7γ1*

All CE7 antibodies, regardless of their isotype, bound to IMR-32 cells as demonstrated in a cell-ELISA using polyclonal isotype-specific labelled antibodies (Figure 1). The titration of CE7 antibodies against a constant number of

**Figure 1a** Binding of CE7γ1 (▲—▲), γ2a (■—■) and γ2b (●—●) to IMR-32 cells. Bound antibodies were measured with isotype-specific β-galactosidase-labelled goat anti-mouse antibodies in ELISA. The data represent mean value of triplicate. Deviations were ⩽15%. **b**, Inhibition of CE7γ1 binding by CE7γ2a and CE7γ2b in an IMR-32 neuroblastoma cell-ELISA. The cells were incubated with dilutions of CE7γ2a or CE7γ2b and a constant amount of CE7γ1. Bound CE7γ1 was measured with β-galactosidase-labelled goat anti-mouse γ1 specific antibody, (■—■) CE7γ1 inhibition by CE7γ2a. (□—□) CE7γ1 inhibition by CE7γ2b.

IMR-32 cells resulted in similar binding curves for all isotypes. Also the binding to the neuroblastoma lines SK-N-AS and SK-N-MC was the same for all isotypes (data not shown). As shown in Figure 1b, by competitive ELISA inhibition, the CE7γ2a and CE7γ2b antibodies recognize the same epitope as the original CE7γ1 antibody and display the same affinity for the neuroblastoma cells.

The cell binding capacity of CE7 antibodies was determined by incubating a constant number of IMR-32 cells with varying amounts of purified, monomeric $^{125}I$-CE7γ1; the Scatchard plot (not shown) was linear with r equal to 0.92. The IMR-32 cells expressed 120,000 binding sites, and the dissociation constant ($K_d$) of the CE7 antibodies was $1.44 \times 10^7\,M^{-1}$.

### *The influence of effector and target cell type*

In preliminary tests, the rates of cell death (spontaneous lysis) of neuroblastoma lines IMR-32, SK-N-AS and SK-N-MC were measured over an 18 h period. The rates of cell death for the three neuroblastomas decreased in the order of: SK-N-MC; SK-N-AS; IMR-32. Based on these results IMR-32 was selected as the target cell for ADCC experiments.

IMR-32 cell survival in the presence of different cell populations of PBMC (effector cells) was measured with and without the addition of antibody (CE7γ2a) in a 6 h assay. The number of IMR-32 target cells added per well was 2,500. Figure 2 summarises the results obtained with mononuclear cells, purified monocytes and nonadherent cells as effectors at E/T ratios of 40:1 and 80:1. With the addition of CE7γ2a antibodies, target cell death was slightly but not significantly increased.

**Figure 2** Time-dependent lysis of IMR-32 target cells in the presence of human mononuclear cells, monocytes and nylon-nonadherent lymphocytes. $^{51}$Cr-labelled IMR-32 (2,500 cells/well) were incubated with and without CE7γ2a (500 ng/well). Effector cells were added at an E/T ratio of 40:1 (□—□) or 80:1 (■—■). Control without CE7γ2a E/T 40:1 (O—O) and 80:1 (●—●). The dotted line refers to antibody-coated target cells in the absence of effector cells (figure at top). Data represent the mean of triplicate.

In contrast to the above, IMR-32 cell survival was distinctly different in the presences of macrophages (Figure 3). In the absence of antibody, the rate of death was very low. With the addition of antibody, target cell killing was markedly increased. The E/T ratios used were 20:1 and 5:1. Pre-exposure of macrophages to rIFN-γ increased both non-specific and specific (antibody-mediated) killing. A post-lysis uptake of $^{51}$Cr by macrophages from the supernatant could be excluded (not shown).

### *ADCC activity of different CE7 isotypes*

Lysis of IRM-32 cells by macrophages or by activated macrophages was determined in the presence of CE7γ1, CE7γ2a or CE7γ2b antibodies, or of a γ2a control anti-arsonate antibody. Figure 4 shows the results of an experiment with an E/T ratio of five and an incubation time of 10 h. Both normal and activated macrophages mediated cell lysis in the presence of CE7γ2a antibodies; neither anti-arsonate at 40 μg ml$^{-1}$, nor any of the other CE7 antibodies showed an effect. Similar results were obtained with other E/T ratios and for other incubation periods, suggesting that macrophage ADCC of the IMR-32 via the CE7 antigen is mediated by the IgG2a isotype exclusively.

### Discussion

In the present study, we analysed anti-neuroblastoma cytolytic effector cells in ADCC assays with CE7γ1, γ2a and γ2b isotype-switch variants, using IMR-32 cells as a target.

**Figure 3** Time-dependent lysis of IMR-32 target cells by human macrophages and rIFN-γ treated macrophages in the presence or absence of CE7γ2a (500 ng well). E/T ratio of 5:1 (□—□) or 20:1 (■—■). Control without antibody E/T 5:1 (O—O) and 20:1 (●—●). The dotted line refers to antibody-coated target without effector. The data represent the mean of triplicate.

**Figure 4** ADCC activities of CE7γ1, CE7γ2a and CE7γ2b and of control IgG2a antibody. Antibody was added at various concentration of $^{51}$Cr-labelled IMR-32. Macrophages and IFNγ activated macrophages at a E/T ratio of 5:1 and antibodies were added. CE7γ2a (■—■) control γ2a (□—□), CE7γ2b (●—●), CE7γ1 (▲—▲. The results are compared to antibody coated IMR-32 cells without effector cells (O—O). Target cell lysis was assessed after 10 h.

Such variants display identical binding characteristics, because they contain the same H chain VDJ and L chain VJ regions attached to different isotypes. Accordingly, different effector functions can be clearly related to the respective isotype, not influenced by differences in antibody affinity and epitope binding. IMR-32 was found to be best suited for this purpose since it showed lowest spontaneous lysis. When measuring cytolysis in a $^{51}$Cr release assay, it was found that different effector cells exerted a significant degree of non-specific activity; these cells were nylon-nonadherent lymphocytes (most probably NK/K cells) and elutriation-purified monocytes. The presence of 'naturally' cytotoxic cells in the monocyte fraction (up to 99% monocytes) was unexpected. If endotoxins were not rigorously excluded, freshly isolated monocytes were found to be cytotoxic in other systems (Ziegler-Heitbrock *et al.*, 1986). During elutriation, monocytes were exposed to amounts of endotoxin sufficient to induce the secretion of TNF-α. Because the sensitivity of our target cells for TNF-α has not been established, TNF-α must be considered as one possible explanation for the observed non-specific lysis. In contrast to the freshly isolated effector cells, monocyte-derived macrophages cultured *in vitro* did not display non-specific lysis.

Macrophage-mediated lysis of IMR-32 targets occurred only with the addition of CE7γ2a antibodies. RIFN-γ treated macrophages enhanced the CE7γ2a-dependent killing of neuroblastoma cells. The γ1 and γ2b isotypes of CE7 did not mediate ADCC. Specificity of cytolytic activity was demonstrated with the addition of an irrelevant γ2a antibody.

Recently, knowledge on human monocyte FcγR has progressed rapidly (Hogg, 1988) and up to now, three types of FcRγ have been found on peripheral blood leukocytes. They are referred to as FcRγI/CD64, FcRγII/CD32 and FcRγIII/CD16, respectively. These FcR are distinguished antigenically, structurally, by isotype binding specificity and by binding affinity (Anderson & Looney, 1986; Fanger *et al.*, 1989). FcRγI, being mainly expressed on cells of the monocyte-macrophage lineage, is unique in that it binds monomeric human IgG1 and IgG3, and murine IgG2a and IgG3. It could therefore be involved in the CE7γ2a-mediated lysis of IMR-32 cells. This is consistent with the findings of Steplewski *et al* (1983) who demonstrated lysis of human colon carcinoma cells by IgG2a-armed monocytoid effectors.

In a study similar to ours, IgG2b was also active in mediating ADCC, although to a lower degree, than IgG2a (Kipps *et al.*, 1985). In their study, unseparated mononuclear cells served as effector cells. Their effector cell preparation contained cells of the NK/K lineage which express the low affinity FcRγIII. Thus, so far the consensus is that γ2a is the most effective murine isotype mediating ADCC regardless of the effector cell involved. The hypothesis that FcRγI mediates lysis of CE7γ2a-covered targets could be proven by the use of blocking antibodies specific for FcRγI; these antibodies were unavailable at the time of this study. In the present study, exposure of macrophages to rIFN-γ, known to upregulate selectively FcRγI/CD64 (Jungi & Peterhans, 1988), promoted enhanced CE7γ2a-mediated lysis (Figure 3).

The antibody CE7, which is one of the few neuroblastoma selective monoclonal antibodies but possesses some unique features (Momoi *et al.*, 1980; Reynolds & Smith, 1982; Cheung *et al.*, 1985; Schönmann *et al.*, 1986), has been used hitherto for diagnostic purposes. For therapeutic use an antibody should not only be specific, but also should mediate appropriate effector functions, such as activation of the complement system and the induction of ADCC. The present study demonstrates that the strategy of selecting isotype switch variants can fulfill these requirements. The γ2a variant of CE7 described here mediates both ADCC and complement fixation while the original γ1 isotype is deficient in both respects.

We gratefully thank CIBA-GEIGY Ltd, Basel, Switzerland for a fellowship to C. d'Uscio and Prof. P. Zahler, Institute of Biochemistry, University of Berne for providing laboratory facilities. The project was sponsored partly by the Swiss and Bernese cancer ligues. The generous gift of recombinant IFN-γ by Biogen, Geneva and the excellent technical assistance in preparation of different types of cells by Mrs M. Brcic, are gratefully acknowledged.

### References

ANDERSON, C.L. & LOONEY, R.J. (1986). Human leukocyte IgG Fc receptors. *Immunol. Today*, **9**, 264.

ANDREESEN, R., PICHT, J. & LÖHR, G.W. (1983). Primary cultures of human blood-born macrophages grown on hydrophobic teflon membranes. *J. Immunol. Methods*, **56**, 295.

CHEUNG, N.K.V., SAARINEN, U.M., NEELY, J.E., LANDMEIER, B., DONOVAN, D. & COCCIA, P.F. (1985). Monoclonal antibodies to a glycolipid antigen on human neuroblastoma cells. *Cancer Res.*, **45**, 2642.

CLEMETSON, K.J., MCGREGOR, J.L., MCEVER, R. & 4 others (1985). Absence of platelet membrane glycoproteins IIb/IIIa from monocytes. *J. Exp. Med.*, **161**, 972.

CONKLING, P., KLASSEN, D.K. & SAGONE, A.L. Jr. (1982). Comparison of antibody-dependent cytotoxicity mediated by human polymorphonuclear cells, monocytes and alveolar macrophages. *Blood*, **60**, 1290.

DE LANDAZURI, M.O., SILVA, A., ALVARES, J. & HERBERMAN, R.B. (1979). Evidence that natural cytotoxicity and antibody-dependent cellular cytotoxicity are mediated in humans by the same effector cell populations. *J. Immunol.*, **123**, 252.

EVANS, A.E., GERSON, J. & SCHNAUFER, L. (1976). Spontaneous regression of neuroblastoma. *Natl Cancer Inst. Monogr.*, **44**, 49.

FANGER, M.W., SHEN, L., GRAZIANO, R.F. & GUYRE, P.M. (1989). Cytotoxicity mediated by human Fc receptors for IgG. *Immunol. Today*, **10**, 92.

FEIT, C., BARTAL, A.H., TAUBER, G., DYMBORT, G. & HIRSHAUT, Y. (1983). An enzyme-linked immunosorbent assay (ELISA) for the detection of monoclonal antibodies recognizing surface antigens expressed on viable cells. *J. Immunol. Meth.*, **58**, 301.

HELLSTRÖM, I., HELLSTRÖM, K.E. & YEH, M. (1982). Lymphocyte-dependent antibodies to antigen 3.1, a cell-surface antigen expressed by a subgroup of human melanomas. *Intl. J. Cancer*, **27**, 281.

HOGG, N. (1988). The structure and function of Fc receptors. *Immunol. Today*, **9**, 185.

HUGHES, M., MARSDEN, H.B. & PALMER, M.K. (1974). Histologic patterns of neuroblastoma related to prognosis and clinical staging. *Cancer*, **34**, 1706.

IMAI, K., PELLEGRINO, M.A., WILSON, B.S. & FERRONE, S. (1982). Higher cytolytic efficiency of an IgG2a than of an IgG1 monoclonal antibody reacting with the same (or spatially close) determination on a human high-molecular-weight melanoma-associated antigen. *Cell. Immunol.*, **72**, 239.

JULIUS, M.H., SIMPSON, E. & HERZENBERG, L.A. (1973). A rapid method for the isolation of functional thymus-derived murine lymphocytes. *Eur. J. Immunol.*, **3**, 645.

JUNGI, T.W. & HAFNER, S. (1986). Quantitative assessment of Fc receptor expression and function during *in vitro* differentiation of human monocytes to macrophages. *Immunology*, **58**, 131.

JUNGI, T.W. & PETERHANS, E. (1988). Change in the chemiluminescence reactivity pattern during *in vitro* differentiation of human monocytes to macrophages. *Blut*, **56**, 213.

JUNGI, T.W., RÜEGG, S.J. & MORELL, A. (1989). Interferon gamma-treated human macrophages display enhanced cytolysis and generation of reactive oxygen metabolites but reduced ingestion upon Fc receptor triggering. *Human Immunol.*, **24**, 77.

KATZ, P., SIMONE, C.B., HENKART, P.A. & FAUCI, A.S. (1980). Mechanism of antibody-dependent cellular cytotoxicity. *J. Clin. Invest.*, **65**, 55.

KEMSHEAD, J.T. & BLACK, J. (1980). Developments in the biology of neuroblastoma: implication for diagnosis and treatment. *Devel. Med. Child Neurol.*, **22**, 816.

KEMSHEAD, J.T., FRITSCHY, J., GOLDMAN, A., MALPAS, J.S. & PRITCHARD, J. (1983). Use of panels of monoclonal antibodies in the differential diagnosis of neuroblastoma and lymphoblastic disorders. *Lancet*, **i**, 12.

KIPPS, T.J., PARHAM, P., PUNT, J. & HERZENBERG, L.A. (1985). Importance of immunoglobulin isotype in human antibody-dependent, cell-mediated cytotoxicity directed by murine monoclonal antibodies. *J. Exp. Med.*, **161**, 1.

MOMOI, M., KENNET, R.H. & GLICK, M.C. (1980). A membrane glycoprotein from human neuroblastoma cells isolated with the use of a monoclonal antibody. *J. Biol. Chem.*, **255**, 11914.

MÜLLER, C.E. & RAJEWSKY, K. (1983). Isolation of immunoglobulin class switch variants from hybridoma lines secreting anti-idiotype antibodies by sequential sublining. *J. Immunol.*, **131**, 877.

PEARSON, G.R. (1978). *In vitro* and *in vivo* investigations on antibody-dependent cellular cytotoxicity. *Contemp. Top. Microbiol. Immunobiol.*, **80**, 65.

RALPH, P., NAKOINZ, I., DIAMOND, P. & YELTON, D. (1980). All classes of murine IgG antibody mediate macrophage phagocytosis and lysis of erythrocytes. *J. Immunol.*, **125**, 1885.

RENTSCH, M., RÖSLER, H., SCHÖNMANN, S.M. & 6 others (1988). *CE7, a Monoclonal Antibody against Human Neuroblastomas: Results of a Preclinical Study*. 18th Internatl. Symposium, Badgastein. Höfer, R. & Bergmann, H. (eds), Schattauer-Verlag.

REYNOLDS, C.P. & SMITH, R.G. (1982). A sensitivie immunoassay for human neuroblastoma cells. In *Hybridomas in Cancer Diagnosis and Treatment*, Mitchell, M.S. & Oettgen, H.F. (eds), Ravens Press: New York, Pp. 235–40.

REYNOLDS, C.P. & MAPLES, J. (1985). Modulation of cell surface antigens accompanies morphological differentiation of human neuroblastoma cell lines. *Adv. Neuroblastoma Res.*, **13**, 37.

SCHÖNMANN, S.M., IYER, J., LAENG, H., GERBER, H.A., KÄSER, H. & BLASER, K. (1986). Production and characterisation of monoclonal antibodies against human neuroblastoma. *Intl J. Cancer*, **37**, 255.

STEPLEWSKI, Z., LUBECK, M.D. & KOPROWSKI, H. (1983). Human macrophages armed with murine immunoglobulin G2a antibodies to tumors destroy human cancer cells. *Science*, **221**, 865.

D'USCIO, C., AMSTUTZ, H.P. & BLASER, K. (1992). Establishment of isotype-switch variants of CE7 anti-neuroblastoma. (in preparation).

ZIEGLER-HEITBROCK, H.W., MÖLLER, A., LINKE, R.P., HAAS, J.G., RIEBER, E.P. & RIETHMULLER, G. (1986). Tumour necrosis factor as effector molecule in monocyte mediated cytotoxicity. *Cancer Res.*, **46**, 5947.